# Faith-Cures

**Published:** 1888-06

**Authors:** 


### FAITH CURES.

A recent number of our contemporary, the Philadelphia *Medical and Surgical Reporter*, brings prominently under notice the latest craze of the nineteenth century. From the report given it appears that in the States the dependence on the supernatural is quite as marked as in the old country, and consequently the mischief proportionately large. It appears that a young married woman in Cincinnati was suffering from a carcinoma, and at the request of her husband, a medical man, the tumor was removed. In a comparatively short time the growth reappeared and grew with great rapidity. The poor patient now became importuned to undergo the "Faith Cure," and her husband was strongly denounced for offering opposition. In short, the case came under the pretentious workers of modern miracles, with the result that the disease steadily gained ground, and finally the patient died, and then followed the regrets that she had not called sooner, that she had shown want of "faith" in first trying rational means, and so forth. When we read such cases we almost despair of the progress of our race ; we seem once more plunged into the intellectual inertia of the middle ages, and find ourselves still swathed with the cerecloths of superstition in an age of telegraphy. Legislation is powerless for good —nothing can open the eyes of the superstitiously blind, except scientific education. Let the rising generation get a good knowledge of science, and legislation is unnecessary. Superstitions fly from the light of science as owls and bats from the light of day.—*Medical Press and Circular.*

